# Integrated Mechanisms of Flavor and Quality Development in Braised Pork: A Study on Volatile Profiles, Texture Dynamics, Nucleotide Catabolism, and Protein Oxidation

**DOI:** 10.3390/foods15030503

**Published:** 2026-02-01

**Authors:** Zhuowen Wang, Jinxuan Cao, Jinpeng Wang, Yuemei Zhang, Wendi Teng, Shuai Zhuang, Ying Wang

**Affiliations:** Key Laboratory of Geriatric Nutrition and Health, School of Food and Health, Beijing Technology and Business University, Beijing 100048, China

**Keywords:** braised pork, tenderness, flavor, stewing, lipid oxidation, protein oxidation

## Abstract

This study aimed to explore the evolution of quality and flavor characteristics of braised pork during the cooking process and clarify the underlying formation mechanisms. Texture analysis revealed that shear force and hardness initially increased during blanching but decreased substantially with an extended stewing time. Low-field NMR indicated a progressive shift in water distribution from immobilized to free states, correlating with cooking loss and tenderness development. GC-MS and E-nose analyses showed significant increases in volatile compound diversity and concentrations, with aldehydes and ketones identified as dominant contributors to the evolving aroma profile. Throughout the processing, an enhancement in protein oxidation and nucleotide degradation was observed. Notably, significant increases were detected in the umami amino acids aspartic acid and glutamic acid, as well as in the umami nucleotide inosine monophosphate (IMP). These changes collectively contributed to the development of the characteristic taste profile. These findings indicate that the superior eating quality evolution and flavor development of braised pork during cooking are governed by the coordinated changes in texture, water distribution, lipid oxidation, and taste-active compounds. The interplay between these factors occurs at different stages of processing, leading to the complex, non-linear enhancement of flavor and texture.

## 1. Introduction

Braised pork is a renowned traditional Chinese dish that is widely appreciated for its distinctive sensory appeal. It presents an appetizing reddish-brown color and a complex, enticing aroma. The dish is characterized by a deeply satisfying, umami-rich, and savory–sweet flavor profile, along with an exceptionally tender, melt-in-the-mouth texture. Despite its relatively high fat-to-lean ratio, it achieves a unique quality of being ‘rich yet not greasy’ while retaining nutritionally relevant components such as proteins and lipids. This harmonious combination of visual, olfactory, gustatory, and textural properties underpins its enduring culinary appeal.

The flavor profile and texture play significant roles in evaluating the quality of braised pork [1,2], which collectively define the multi-dimensional sensory experience of the final product, further influencing consumer acceptance and preference. For the food industry and large-scale production, precise control and standardization of these attributes are important. Therefore, a mechanistic understanding of how these properties evolve during thermal processing is fundamental to advancing product optimization and manufacturing control. Several studies have demonstrated that the processing conditions and formulation play critical roles in shaping the flavor and textural attributes of braised pork. For example, sous-vide cooking has been reported to improve flavor development and digestibility while modifying textural properties [3], and E-nose combined with SPME-GC-MS has been used to reveal that the seasoning composition markedly influences volatile profiles [4]. Yao et al. proposed a novel gradient-temperature heating method aimed at improving the textural properties of braised pork [5]. Other studies have examined the effects of processing factors on flavor and texture development, including the influence of seasoning addition on the formation of characteristic aroma compounds, where seasoning incorporation was found to effectively inhibit lipid oxidation while promoting the Maillard reaction [6]. In addition, correlations between sensory textural attributes and physicochemical properties have been explored to enable a more comprehensive and objective evaluation of the braised pork texture [7]. The development of these ideal characteristics constitutes a dynamic and complex process during prolonged stewing, involving a series of biochemical and physical transformations. Despite centuries of refinement in the culinary tradition of braised pork, an integrated understanding of how the texture, water distribution, biochemical changes, and flavor evolution interact during cooking remains limited.

To address this knowledge gap, this study focuses on investigating the complex interplay among key factors—texture, flavor, and physicochemical changes—during the thermal processing of braised pork. While prior studies have explored the impact of individual factors such as cooking methods, seasoning, and lipid oxidation, a comprehensive, integrated understanding of how these factors collectively evolve during braising remains fragmented. This work aims to fill that gap by providing a systematic, multidimensional analysis of the thermal process and its effects on the sensory properties of braised pork. By understanding the underlying mechanisms driving flavor development, texture enhancement, and moisture retention, this study will offer practical insights into improving cooking techniques for both industrial and consumer applications. The findings are expected to advance both scientific knowledge and industrial standardization of braised pork production.

## 2. Materials and Methods

### 2.1. Materials and Samples

To ensure raw material uniformity, standard white pork belly from the lower abdomen (weight: 90–100 kg, approximately 7 months old) was sourced from the Pengcheng Food Branch of Beijing Shunxin Agriculture Co., Ltd. (Beijing, China). The pork (500 g) was rinsed, cut into 3 cm × 3 cm × 4 cm cubes, and placed in an iron pot with 1000 g of purified water. The traditional braised pork samples were prepared as follows: the meat was heated and boiled at 2200 W for 5 min. After skimming off the scum, it was removed, rinsed, and replenished with 2500 mL of water. Then, seasonings were added: 25 g ginger, 20 g scallion (both purchased from Yonghui Supermarket), 10 g salt (Zhongshi salt, a natural sea salt), 25 g sugar (Jingshi sugar, pure white granulated sugar), 20 g soy sauce (Lin Jinji, a mushroom soy sauce), and 25 g cooking wine (Wang Zhihe, refined cooking wine). The pot was heated again at 2200 W until boiling, and then, the power was reduced to 1000 W for stewing. To investigate the evolution and formation mechanisms of flavor and tenderness in braised pork, samples from different processing stages were designated as follows: the Control group (raw pork), the B0 group (blanched for 5 min), the B1 group (stewed for 30 min), the B2 group (stewed for 60 min), and the B3 group (stewed for 120 min). All treatment groups—Control, B0, B1, B2, and B3—were prepared from the same batch of pork belly. At each time point, representative pork belly (approximately 100–110 g) was removed from the pot as a sub-sample for subsequent analysis.

After heating, all samples were rapidly cooled by immersing the container in an ice-water bath until the core temperature reached 25 °C (ambient room temperature), and surface seasonings were carefully removed and stored at −80 °C until further analysis.

### 2.2. Texture Profile Analysis (TPA)

The texture analysis was performed according to the method described by Gao et al. [8], with minor modifications. After cooling to room temperature, the lean meat samples were cut into cubes with dimensions of approximately 1 cm^3^. The texture of these cubes was then measured using a texture analyzer (TA. portable, Bosin Tech, Beijing, China) equipped with a P50 probe. The measurement parameters were set as follows: pre-test speed, 1.0 mm/s; test speed, 0.5 mm/s; post-test speed, 1.0 mm/s; compression ratio, 30%; trigger force, 5 g. For each treatment group, measurements were performed on three independent 1 cm^3^ meat samples (biological replicates), with each sample measured once under identical conditions. The entire experiment was repeated independently three times. The results from all replicates were averaged for final analysis.

### 2.3. Shear Force Analysis

The lean meat samples were cut into cubes measuring 1 cm^3^ for shear force determination. For this measurement, the texture analyzer (TA. portable, Bosin Tech, Beijing, China) was fitted with a rectangular blade. The shear test was performed by applying the blade perpendicular to the orientation of the muscle fibers. The maximum force applied during each test was recorded. Experimental parameters were set as follows: pre-test speed, 1.00 mm/s; mid-test speed, 0.50 mm/s; post-test speed, 1.00 mm/s; compression ratio, 90%; trigger force, 5 g. Measurements were performed on three independent 1 cm^3^ meat samples (biological replicates), with each sample measured once under identical conditions. The entire experiment was repeated independently three times. The results from all replicates were averaged for final analysis.

### 2.4. Water-Holding Capacity Analysis

The centrifugation loss was determined according to the procedure described by Li et al. [9], with minor modifications. Approximately 2 g of the lean meat sample was blotted with absorbent paper to remove surface moisture and then transferred to a centrifuge tube. The sample was centrifugated at 4000 rpm for 15 min at 25 °C. Subsequently, the sample was retrieved and accurately weighed. The centrifugation loss (%), which inversely reflects the water-holding capacity (WHC) of meat, was calculated using the following Formula (1):

(1) WHC (%) = [(*W*_0_ − *W*_1_)/*W*_0_] × 100

where *W*_0_ and *W*_1_ represent the weight of the lean meat before and after centrifugation, respectively.

### 2.5. Low-Field 1H NMR Measurements (LF-NMR)

The water status in the lean part of pork belly at different processing stages was determined and analyzed using low-field nuclear magnetic resonance (LF-NMR) [10]. The lean meat of the pork belly samples was cut into small pieces measuring 2.0 × 1.0 × 0.5 cm. These pieces were then placed inside a 25 mm diameter cylindrical glass tube positioned at the magnet center. The transverse relaxation properties of the samples were measured using the Carr–Purcell–Meiboom–Gill (CPMG) pulse sequence. The experimental parameters were set as follows: number of sampling points, 125,030; number of scan repetitions, 16; number of echoes, 2000. The experiment was performed in triplicate, and the results were averaged.

### 2.6. Surface Hydrophobicity Analysis

The surface hydrophobicity of proteins was determined based on the binding capacity of hydrophobic groups to bromophenol blue (BPB) [11]. The lean meat sample was minced, after which 2 g of the sample was combined with 20 mL of phosphate-buffered saline (PBS; 20 mmol/L, pH 6.0) and homogenized at 20,000 rpm for 30 s using a high-speed homogenizer. The protein concentration of the resulting suspension was adjusted to 5 mg/mL. Aliquots (1 mL each) of the sample suspension and PBS (control) were separately mixed with 200 μL of BPB solution (1 mg/mL). After stirring in the dark at room temperature for 10 min, centrifugation was performed at 2500 × *g* for 15 min at 4 °C. The resulting supernatant was diluted 10-fold with PBS, and the absorbance at 595 nm was measured using a UV-Vis spectrophotometer. The BPB-binding capacity (hydrophobicity index) was calculated according to the following Formula (2):

(2) BPB binding capacity (μg) = 200 × (Ac − As)/Ac

where Ac and As represent the absorbance of the control and the sample, respectively.

### 2.7. Total Carbonyl and Sulfhydryl Contents

The total carbonyl content was determined using 2,4-dinitrophenylhydrazine (DNPH) according to the method of Mercier et al. [12], with minor modifications. The myofibrillar protein (MP) solution was diluted to a concentration of 2 mg/mL with 100 mmol/L KCl solution. Two aliquots (0.5 mL each) of the protein solution were then dispensed into separate test tubes. To one aliquot, 0.5 mL of 2 mol/L HCl containing 0.2% (*w*/*v*) DNPH was added, whereas 0.5 mL of 2 mol/L HCl (without DNPH) was added to the other aliquot as the control group. Both mixtures were incubated at room temperature in the dark for 1 h, with vortex mixing for 1 min at 10 min intervals to ensure a thorough reaction. Subsequently, 0.5 mL of a 20% (*w*/*v*) trichloroacetic acid (TCA) solution was added to each tube to precipitate the proteins. The samples were then centrifuged at 10,000 × *g* for 5 min. The resulting pellets were washed three times with 2 mL of an ethyl acetate-ethanol mixture (1:1, *v*/*v*) and then redissolved in 3 mL of 6 mol/L guanidine hydrochloride, followed by incubation in a 37 °C water bath for 30 min. After centrifugation at 8000 × *g* for 5 min, the absorbance of the collected supernatant was measured at a wavelength of 370 nm. The carbonyl content was calculated using the following Formula (3):

(3) Carbonyl content (nmol/mg protein) = (3,000,000 × A_1_)/(0.5 × ε_1_ × ρ)

where A_1_ is the absorbance at 370 nm, ρ is the protein concentration (mg/mL), and ε_1_ is the molar extinction coefficient (22,000 L·mol^−1^·cm^−1^).

The total sulfhydryl group content was determined following the method described by Cao et al. [13], with slight modifications. Briefly, 100 μL of the protein sample was mixed with 1 mL of Tris-glycine buffer (0.086 mol/L Tris, 0.09 mol/L glycine, 4 mmol/L EDTA, pH 8.0). Then, 20 μL of Ellman’s reagent (4 mg of 5,5′-dithiobis-(2-nitrobenzoic acid) (DTNB) dissolved in 1 mL of Tris-glycine buffer) was added. The mixture was vortexed thoroughly and incubated in a water bath at 25 ± 1 °C for 1 h. After centrifugation at 12,000 × *g* for 10 min, the supernatant was collected. The absorbance of the supernatant was measured at 412 nm, using a control (DTNB dissolved in complete protein extraction buffer as the blank reference). The sulfhydryl group content was calculated using Equation (4):

(4) Sulfhydryl content (μmol/g protein) = 73.53 × A_412_ × D/ρ (1)

where 73.53 = 10^6^/(1.36 × 10^4^), 1.36 × 10^4^ is the molar extinction coefficient (L·mol^−1^·cm^−1^), D is the dilution factor, and ρ is the protein concentration of the sample (mg/mL).

### 2.8. TBARS (Thiobarbituric Acid Reactive Substance) Analysis

The TBARS value was determined based on the reference with modifications [14]. The sample (5.0 g) was homogenized with 25 mL of 7.5% trichloroacetic acid solution at 10,000 rpm for 30 s. The homogenate was filtered, and 5 mL of filtrate was mixed with 5 mL of 0.02 mol/L thiobarbituric acid solution. After thorough vortexing, the mixture was heated in a water bath at 100 °C for 40 min and then cooled for 1 h. The cooled mixture was centrifuged at 5000× *g* for 5 min at 4 °C. The supernatant was then collected and extracted with 1.0 mL chloroform. The upper layer was carefully separated for analysis. The absorbance of each sample was measured at 532 nm and 600 nm using 96-well plates. The TBARS value was calculated using the following formula: TBARS (mg/kg) = (A_532_ − A_600_)/155 × 72.06 × 1000/10. The values were expressed as mg of malondialdehyde per kg of muscle. All determinations were performed in triplicate.

### 2.9. GC-MS Analysis

The volatile compounds of braised pork at different stages were extracted and quantified via headspace solid-phase microextraction gas chromatography–mass spectrometry (HS-SPME-GC-MS). GC-MS analysis was performed using an Agilent G2790A GC-MS system (Agilent Technologies, Santa Clara, CA, USA).

Following the method of Wang et al. [15], 5.0 g of homogenized sample was placed into a 20 mL headspace vial. Then, 10 μL of a 2-methyl-3-heptanone internal standard solution (0.078 g/L) was added, and the vial was immediately sealed. After equilibration for 15 min, volatile compounds were collected using a 65 μm of PDMS/DVB fiber via automated solid-phase microextraction (SPE) at 60 °C for 30 min. Finally, the analytes were thermally desorbed in the GC–MS injector for 10 min.

Chromatographic separation was performed using an HP 5MS capillary column (30 m × 0.25 mm, 0.25 μm) with helium as the carrier gas at a constant flow rate of 1.5 mL/min in splitless mode. The injector temperature was maintained at 250 °C. The oven temperature program was as follows: initial temperature, 40 °C, held for 3 min, increased at 5 °C/min to 90 °C, then raised at 10 °C/min to 230 °C, and held for 7 min.

Mass spectrometric detection employed electron ionization (70 eV) with an ion source temperature of 200 °C and an interface temperature of 250 °C. The filament current was set to 200 μA and the detector voltage to 1.0 kV. Data were acquired in the mass range of 35–350 *m*/*z*.

Volatile compounds were identified by comparing mass spectra with the NIST 08/08s libraries (match factor > 80). Quantification was based on the internal standard method using the following Equation (5):

(5) Xi = Ai/As× Cs

where Xi denotes the mass concentration (g/L) of the target analyte, Cs represents the mass concentration (g/L) of the internal standard (2-methyl-3-heptanone), and Ai and As correspond to the peak area of the target analyte and the internal standard, respectively.

### 2.10. Electronic Nose Analysis

A selective electronic nose system was used to monitor the evolution of the odor distribution during the thermal processing of braised pork. Sample analysis was conducted using an electronic nose (E-nose) system (PEN3, AIRSENSE Analytics, Germany) featuring ten metal oxide semiconductor (MOS) sensors with defined specificities: W1C (aromatic compounds), W5S (nitrogen oxides), W3C (ammonia and aromatic compounds), W6S (hydrogen), W5C (olefins and aromatic compounds), W1S (methane and hydrocarbons), W1W (sulfides), W2S (alcohols and aromatic compounds), W2W (aromatic and organic sulfides), and W3S (olefins).

The electronic nose analysis was carried out according to the method described by Zhao et al. [16], with minor modifications; 5.0 ± 0.1 g of homogenized sample was placed into a standardized glass sampling container and equilibrated at ambient temperature (25 ± 1 °C) for 60 min. Prior to analysis, the sensor array was calibrated by purging with clean air at a flow rate of 1.0 L/min for 30 min to ensure a stable baseline. Each measurement cycle consisted of a 150 s sensor sampling period and a 120 s sensor cleaning period, with a constant gas flow of 1.0 L/min maintained throughout. All thermal processing stages were repeated three times, and the average sensor responses were used for subsequent data analysis.

### 2.11. Free Amino Acid Analysis

The free amino acid composition was analyzed following the method of [17] with modifications. Briefly, 5.00 ± 0.01 g of sample was homogenized (10,000 rpm, 1 min) with 15 mL of 5% (*w*/*v*) trichloroacetic acid solution using a high-speed homogenizer. The homogenate was allowed to stand for 2 h at room temperature (25 ± 1 °C) for complete protein precipitation, followed by centrifugation at 10,000× *g* for 30 min at 4 °C. The resulting supernatant was filtered through a 0.22 μm aqueous-phase membrane filter (Millipore, St. Louis, MI, USA) prior to analysis. Amino acid quantification was performed on an Agilent 1260 HPLC system (Agilent Technologies, USA) equipped with a UV-Vis detector, using an automated online o-phthaldialdehyde (OPA) derivatization. Separation was carried out on a ZORBAX Eclipse AAA column (4.6 × 150 mm, 3.5 μm) maintained at 40 °C. The analysis used a detection wavelength of 338 nm and a mobile phase gradient between the buffer (phase A, 10 mM sodium phosphate dibasic and 10 mM sodium borate, pH = 8.2) and an organic mixture (phase B: acetonitrile/methanol/water, 45:45:10, *v*/*v*/*v*) at a flow rate of 1.0 mL/min. The gradient started at 2% B, increased to 57% B by 13.4 min, then to 100% B by 13.5 min (held until 15.7 min), and was finally brought back to 2% B for column re-equilibration. The injection volume was 3 μL.

### 2.12. Nucleotide Analysis

Nucleotides were extracted and analyzed using the following procedure. First, 4.00 ± 0.05 g of a thoroughly mixed sample was homogenized with 20 mL of pre-cooled 5% (*v*/*v*) perchloric acid in an ice-water bath (10,000 rpm, 30 s). After centrifugation at 8000 × *g* for 10 min at 4 °C, the supernatant was collected using filter paper. The remaining pellet was re-extracted with 10 mL of 5% perchloric acid under the same conditions. The combined supernatants were adjusted to pH 6.5 ± 0.1 using 1 M potassium hydroxide and then diluted to a final volume of 50 mL with HPLC-grade water. Prior to HPLC analysis, 1 mL aliquots were centrifuged again (8000× *g*, 10 min, 25 °C) and filtered through 0.22 μm nylon membranes.

HPLC analysis (Agilent Technologies, USA) was carried out using a Polaris 5C18-PAQ column maintained at 30 °C, with UV detection at 254 nm. An isocratic elution was applied for 28 min using 0.05 M phosphate buffer (pH 6.5) as the mobile phase at a flow rate of 1.0 mL/min. The injection volume was 20 μL.

### 2.13. Statistical Analysis

All data are presented as the mean ± standard deviation. The experimental unit was defined as an independent cooking process. For each treatment (processing stage), three independent biological replicates were obtained from three separate cooking experiments performed on different days using fresh raw materials. Within each biological replicate, samples were collected at the same processing stage and subdivided for the different analytical assays. Instrumental analyses were performed with technical replicates where applicable.

Statistical differences were evaluated using one-way analysis of variance (ANOVA) in IBM SPSS Statistics 26, and differences among means were further compared using Tukey’s honestly significant difference (HSD) post-hoc test, with a significance level set at (*p* < 0.05). Relationships between variables were assessed using Pearson correlation analysis with the same software. All figures were prepared using Origin 2021 software.

## 3. Results and Discussion

### 3.1. Changes in Lean Meat Tenderness During the Cooking of Braised Pork

Tenderness is a critical quality attribute of meat products, substantially influencing consumer preference and acceptance. As presented in Table 1, the changes in tenderness of the lean meat in braised pork during stewing were assessed by measuring shear force and texture profile analysis (TPA) parameters [18]. It is widely accepted that a lower shear force generally corresponds to higher tenderness [19]. In raw meat, the initial shear force was relatively low; however, it increased significantly during the first 5 min of blanching. Subsequently, the shear force of the lean meat decreased sharply throughout the stewing process. Similarly, hardness exhibited a pattern of an initial significant increase followed by a decrease with rising temperatures and prolonged time. Specifically, compared with the blanched stage (B0), shear force decreased by approximately 57% at the end of stewing (B3), while hardness was reduced by nearly 70%, indicating a substantial improvement in tenderness during prolonged thermal treatment. Cohesiveness, springiness, and chewiness showed trends analogous to that of hardness. Collagen, the primary component of connective tissue, undergoes complete gelatinization at temperatures above 65 °C [20]. Consequently, the denaturation of collagen may lead to a two-stage process: contraction of connective tissue during the initial heating stage, followed by its gelatinization and dissolution at elevated temperatures in the second stage [21]. Consistent with our findings, a stewing duration of 30 min emerged as a critical phase for the development of tenderness in lean meat. During the initial processing stage (0–5 min), tenderness generally decreased. In contrast, with an extended stewing time (>30 min), both shear force and hardness dropped markedly, leading to a significant improvement in tenderness, a trend consistent with the report by Wang et al. [22].

During the initial 5 min of blanching, as the temperature increased from 25 °C to 100 °C, the decline in tenderness was primarily attributed to the denaturation of myofibrillar proteins. This process induced the contraction of muscle fibers and a reduction in the water-holding capacity [23]. Structural changes in myofibrillar proteins during heating exerted a significant influence on tenderness [24]. When the temperature exceeded the denaturation threshold, the majority of proteins transitioned from their native state to a denatured state. With further prolongation of the cooking time, the gradual degradation of muscle tissue and the intensified disruption of the myofibrillar protein structure collectively contributed to the progressive softening of the meat. This softening was likely attributed to the thermal-induced dissociation of protein structures and the breakdown of muscle tissue [25]. These results indicated that the lean meat at the 5 min blanching stage exhibited the highest chewing resistance and least palatable texture, while tenderness progressively improved with the stewing time, reaching significantly enhanced edibility after 30 min of stewing.

### 3.2. Water-Holding Capacity

**Figure 1:**
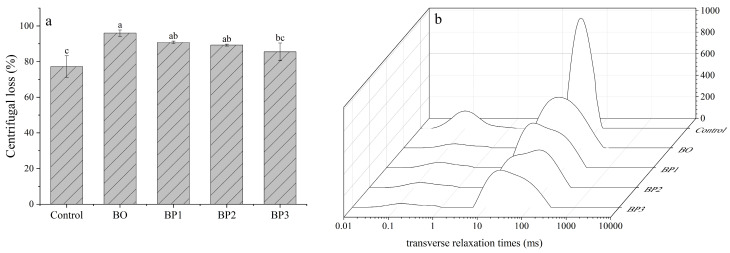
Centrifugal loss (%) (**a**) and LF-NMR parameters (**b**). Data are expressed as the mean ± standard deviation (n = 3); different letters indicate significant differences (*p* < 0.05), as determined via one-way analysis of variance (ANOVA) followed by Tukey’s multiple comparison test.

Centrifugal loss, which reflects the water-holding capacity of meat under external force, was found to directly affect its tenderness [26]. As shown in Figure 1a, the cooked meat exhibited higher centrifugation loss than the fresh raw meat, which was consistent with the findings reported by Wang et al. [27]. During processing, the centrifugation loss showed a trend of an initial increase, peaking at the B0 stage, followed by a gradual decrease. This trend was consistent with the variations in the shear force and hardness of the lean meat. The observed increase in centrifugation loss at the early stage (B0) may have been primarily due to the denaturation and structural contraction of myofibrillar proteins, which can drastically reduce their water-binding capacity. Alternatively, it could also be due to the reduction in inter-myofibrillar spaces, which physically expelled water from the myofibrils matrix [28]. The pronounced decrease in centrifugation loss after 30 min of stewing could be explained by the lower proportion of undenatured proteins, which reduced the amount of releasable water. Prior studies indicated that the formation of a dense myofibrillar gel network during stewing contributed to enhanced water-holding capacity (WHC), thereby resulting in reduced centrifugal loss [8]. Furthermore, the conversion of collagen to gelatin can absorb additional moisture, thereby reducing water loss. Moreover, the coagulation of denatured protein molecules between fibers may increase the tortuosity of water migration pathways, thereby impeding moisture migration and lower centrifugation loss [22]. Therefore, the centrifugation-loss results indicated the water-retention behavior of the lean meat in braised pork during cooking and ultimately its influence on meat tenderness.

### 3.3. Water Distribution and Mobility of Lean Meat Characterized via LFNMR

As shown in Figure 1b, the immobilized water content was highest in the raw meat. Low-field nuclear magnetic resonance (LF-NMR) relaxometry provides direct information into water–protein interactions and quantifies the binding states and distribution of water within a sample. Generally, three types of water populations are identified in muscle based on their transverse relaxation times (T_2_) [29]: (a) T2b (0–10 ms), corresponding to water tightly bound to macromolecules, referred to as bound water; (b) T_21_ (10–100 ms), attributed to water entrapped within the myofibrillar network, known as immobilized water; and (c) T_22_ (100–1000 ms), representing water located in the extracellular spaces between myofibrils, termed free water.

As processing progressed, the relative peak area corresponding to immobilized water decreased, while that of free water increased, indicating a redistribution of water from immobilized to more mobile states. The gradual diminution of the T2b peak suggests alterations in the water mobility and binding status during thermal processing, rather than direct quantification of the total moisture content. With an increasing temperature, the peak areas for both bound water and immobilized water continued to decline, whereas the peak area for free water showed a significant elevation. Studies have indicated that T_21_ is closely associated with protein changes during heating. The denaturation and contraction of myofibrillar proteins during this process expel immobile water, which reduces juiciness and adversely affects tenderness [30]. Among the relaxation components, T_21_ exhibited the most pronounced changes in both the peak position and area, with its signal intensity declining as the heating temperature increased. The increase in the free water peak area could be interpreted as an increase in the volume of extra-myofibrillar water, which was consistent with the observed changes in centrifugation loss and meat tenderness. The water redistribution resulted from myofibrillar contraction at elevated temperatures, which drove water from the organized myofibrillar matrix into extra-myofibrillar spaces, ultimately leading to cooking loss as water migrated to the meat surface. Previous research demonstrated that the water-holding capacity of meat is highly correlated with the mobility of water within the highly organized myofibrillar protein matrix and the content of extra-myofibrillar water [30]. Therefore, water present in the free state represents the potential form contributing to cooking loss. The formation of and increase in free water during heating could thus be a primary driver of water loss.

### 3.4. Changes in Tertiary Structure of Proteins Analyzed Based on Surface Hydrophobicity

**Figure 2:**
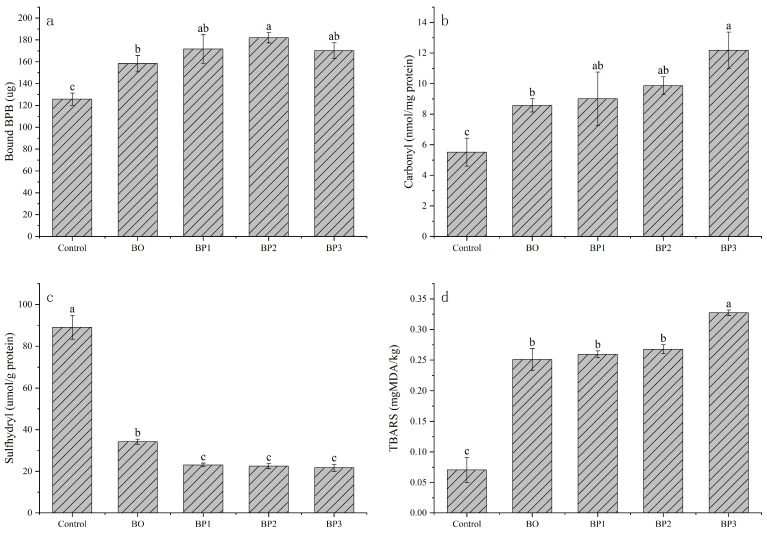
Surface hydrophobicity (**a**), carbonyl content (**b**), sulfhydryl content (**c**), and TBARS value (**d**). Data are presented as the mean ± standard deviation (n = 3); different letters indicate significant differences (*p* < 0.05), as determined via one-way analysis of variance (ANOVA) followed by Tukey’s multiple comparison test.

Conformational changes in proteins during heating were likely associated with alterations in water distribution within the muscle and were also a key factor contributing to changes in tenderness. Changes in the tertiary structures of proteins can be assessed by measuring surface hydrophobicity. Generally, hydrophobic amino acid residues buried within the protein structure become exposed upon heat treatment due to molecular unfolding. This leads to an increase in surface hydrophobicity, thereby promoting stronger hydrophobic interactions during gel formation [31]. As shown in Figure 2a, The surface hydrophobicity was low in raw meat but increased significantly after processing (*p* < 0.05). This increase was likely due to the heat-induced disruption of hydrogen and ionic bonds, which led to conformational alterations and the destabilization of protein structures. Consequently, the exposure of internal hydrophobic residues altered the distribution of hydrophobic domains on the protein surface, resulting in the observed increase in surface hydrophobicity. This finding was consistent with the results reported by J. et al. [32]. A higher surface hydrophobicity indicated a greater number of hydrophobic groups on the protein surface and, consequently, a higher degree of denaturation. This result suggested that the number of exposed hydrophobic residues increased with a rising temperature during the initial heating stage [33]. Furthermore, the enhancement of surface hydrophobicity resulting from protein denaturation reduced the water-binding capacity of proteins. This reduction could also explain the decrease in bound water observed during the initial heating phase.

### 3.5. Changes in Carbonyl and Sulfhydryl Contents Associated with Protein Oxidation

Protein oxidation has been reported to be associated with reduced meat tenderness [34]. Total carbonyl and sulfhydryl group contents were measured to evaluate protein oxidation during the cooking of braised pork, as shown in Figure 2b,c. The results demonstrated that protein carbonyl content increased significantly during processing, whereas the protein sulfhydryl content decreased markedly from the Control to BP1 stage and subsequently remained relatively stable from BP1 to BP3.

Carbonylation is a form of post-translational modification involving reactive oxygen species. Most protein oxidation occurs on the side chains of amino acid residues. Oxidation can induce structural alterations in proteins, including aromatic hydroxylation, sulfoxidation, and the introduction of carbonyl groups [35]. Generally, a higher carbonyl concentration indicates a greater extent of protein oxidation [36]. As shown in the figure, the initial carbonyl content was 5.52 nmol/mg protein, which increased to 12.18 nmol/mg protein in the final product. Previous studies showed that carbonyl formation alters the conformation of myofibrillar proteins, induces denaturation, and leads to a loss of functionality [37]. The sulfhydryl group concentration in braised pork decreased from 89.09 μmol/g protein in the raw meat to 21.75 μmol/g protein in the final product. This decrease was attributed to enhanced protein oxidation during high-temperature stewing and the oxidation of sulfhydryl groups to form disulfide bonds upon heating, which was consistent with literature reports [38]. This oxidation could further lead to the formation of intermolecular or intramolecular protein cross-links [39], which resulted in tighter packing of myofibrils and exacerbated muscle contraction [40], ultimately affecting the water-holding capacity of the meat.

### 3.6. Changes in TBARS Associated with Lipid Oxidation

The formation of TBARS serves as an indicator of lipid oxidation. This process alters the flavor, nutritional value, and shelf-life of food products by generating secondary oxidation products, with malondialdehyde (MDA) being a primary marker [41]. Figure 2d demonstrates a clear positive correlation between the processing time and TBARS values, indicative of progressive lipid oxidation during thermal processing. Raw meat exhibited the lowest TBARS concentration (0.07 mg MDA/kg), which increased significantly to 0.25 mg MDA/kg after 5 min of blanching and further rose to 0.33 mg MDA/kg following extended stewing. This trend aligns with established patterns observed in poultry and other meat, where prolonged cooking durations promote the accumulation of secondary oxidation products such as malondialdehyde [42,43]. The thermal energy input during cooking during prolonged cooking is recognized as a primary driver of lipid oxidation in meat matrices, accelerating the degradation of unsaturated fatty acids and consequent formation of TBARS [44]. The primary substrates for oxidation in pork belly are likely the phospholipids and triglycerides rich in polyunsaturated fatty acids (PUFAs). Their oxidation proceeds via a free radical chain mechanism, thermally accelerated, leading to the formation of various volatile aldehydes and ketones (some detected in our GC-MS analysis) that contribute to the characteristic cooked aroma [45].

### 3.7. GC-MS Analysis of Flavor Compounds

**Figure 3:**
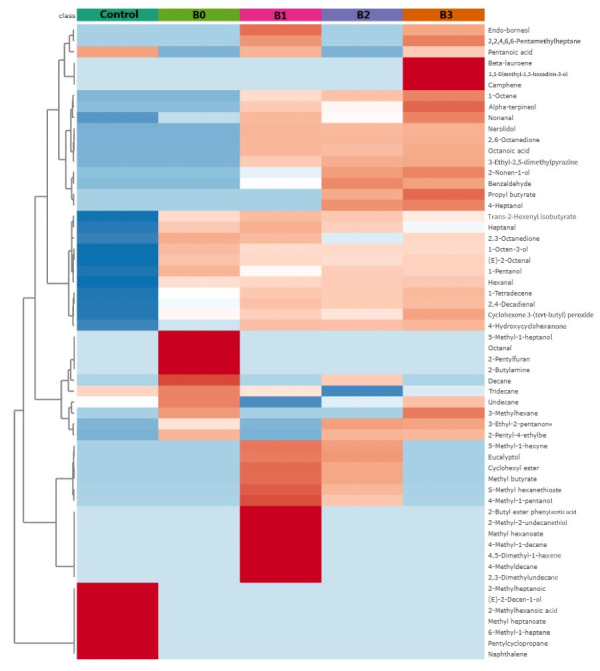
Heat map of volatile compound distribution in braised pork at different processing stages, based on GC-MS analysis.

The volatile flavor compounds of braised pork at different processing stages were dynamically monitored using gas chromatography–mass spectrometry (GC–MS). As shown in the Table A1 and Figure 3, only 11 compounds were identified in the raw meat, comprising two aldehydes, three acids, one ester, one alkene, three alkanes, and one heterocyclic compound. The profile was characterized by low compound diversity and low overall relative abundance of volatile compounds. Following blanching, both the diversity and concentration of flavor compounds increased markedly: 22 compounds were identified, with a total content of 517.64 μg/kg. The stewing process constituted the primary stage for the formation of volatile flavor compounds in braised pork. The first stewing stage (B1) showed the highest variety, with 39 compounds identified and a total content of 1123.95 μg/kg, indicating substantial generation of flavor substances at this point. In the final product, 33 flavor compounds were identified, reaching the highest total content of 1456.32 μg/kg observed throughout processing. Overall, processing significantly enhanced both the diversity and total abundance of flavor compounds in braised pork.

Aldehydes primarily originate from the oxidative degradation of fatty acids. Due to the auto-oxidation of fat in pork belly [46], a small amount of aldehydes was detected even in raw meat, albeit limited to only two types and at low concentrations. After blanching, six aldehydes were detected (334.71 μg/kg), and their content increased progressively with the processing time, indicating a continuous intensification of lipid oxidation during processing. Hexanal and nonanal were present throughout the entire processing with relatively high concentrations, making them major contributors to flavor. Hexanal has been confirmed as a principal aldehyde produced during lipid oxidation in meat [47] and imparts fatty and grassy notes. 2,6-Octadienal, benzaldehyde, and neral began to form during the stewing stages. Among these, 2,6-octadienal has been identified as a characteristic aroma compound of braised pork. Benzaldehyde and neral were likely derived from seasonings such as ginger, possessing citrus notes. Aldehydes derived from lipid oxidation can participate in the Maillard reaction during the later stages of thermal processing. The interaction between the Maillard reaction and lipid oxidation products contributes to the characteristic cooked flavor of meat products [48], enhancing the aroma complexity and harmony of the aroma.

Alcohols fluctuated in content during heating. However, due to their generally high odor thresholds, their impact on the overall aroma quality was limited [49]. No alcohols were detected in raw meat. After blanching, three alcohols were detected with a total content of 49 μg/kg. The highest variety of alcohols was observed in the first stewing stage (B1), while the highest alcohol content was found in the final product (51.57 μg/kg). 1-Octen-3-ol, which imparts a cooked mushroom odor, has been identified as a major alcoholic compound in braised pork and is derived from the β-oxidation of linoleic acid [50]. Eucalyptol and α-terpineol, which appeared in the first stewing stage, are likely derived from spices.

Ketones are also products of lipid oxidation, primarily generated through the oxidation of fatty acids or the catabolism of amino acids. Due to their distinctive flavors, ketones are considered to influence the flavor profile of meat products [49]. No ketones were present in raw meat. The blanched sample contained three ketones with a content of 39.88 μg/kg. The total ketone content reached its peak of 258.47 μg/kg in the final product. Ketones consistently detected across processing stages with relatively high concentrations included 4-hydroxycyclohexanone, 2,3-octanedione, and 3-ethyl-2-pentanone. These are regarded as the primary sweet-like compounds in braised pork samples [50]. The number of volatile ketones increased with a rising processing temperature, a trend that is consistent with previous studies [51].

Esters form predominantly via esterification reactions and interactions among various products, and they generally possess low odor thresholds [52]. One ester was present in raw meat, while two were detected in the final product, with a relatively low total content of 4.76 μg/kg. The ester content peaked in the first stewing stage (B1) at 40.28 μg/kg. The subsequent decline was likely due to further reactions, degradation, and interconversion during later thermal treatment stages, leading to a gradual decrease in the concentration [46]. Methyl butyrate, cyclohexyl ester, hexyl formate, and S-methyl hexanethioate were absent in raw and blanched meat but were present in stewed samples. This indicated that these compounds were formed during the stewing process and contributed to the flavor of braised pork. Ester compounds typically impart fruity, fresh, and sweet aromas, adding a distinctive note to the flavor of braised pork.

Hydrocarbons, mainly alkanes and alkenes, exhibited relatively high variety and content during the stewing stages, reaching 155.62 μg/kg in the final product. The dominant hydrocarbon types varied across different stages. More alkenes, such as myrcene (pleasant balsamic odor) and camphene (camphoraceous odor), were detected in the final product. Compounds including myrcene, camphene, β-phellandrene, and β-pinene are likely derived from ginger and other spices. These results indicated that the addition of seasonings such as ginger, garlic, and chili pepper provided diverse sources of volatile compounds to the pork belly, thereby enhancing the overall richness of the aroma.

Heterocyclic compounds, primarily pyrazines, furans, and furfural, are typical products generated from the degradation and reactions of amino acids, peptides, and proteins under thermal conditions. 2-Pentylfuran, generated during the stewing stages, is likely to contribute significantly to the Maillard reaction. 2-Pentylfuran is characterized by fruity and metallic notes, has a low perception threshold, originates from the oxidation of linoleic acid, and has been reported in various cooked meat samples [53]. A gradual increase in 3-ethyl-2,5-dimethylpyrazine was observed, a trend consistent with findings reported in roasted lamb [54]. However, the total concentration of these Maillard reaction products did not increase continuously with an extended heating time. This phenomenon may be attributed to their role in altering the final equilibrium of flavor compounds, thereby influencing the overall flavor profile. Consequently, the changes in the content of certain compounds did not follow a uniform trend [55].

### 3.8. Electronic Nose Analysis

**Figure 4:**
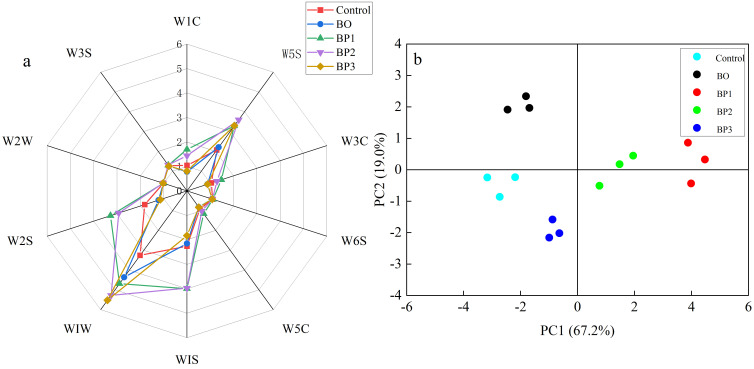
Electronic nose response values (**a**) and PCA plot (**b**).

The electronic nose (E-nose), designed to simulate human olfactory perception, offers superior capability over a sensory evaluation in identifying subtle differences among samples. As shown in Figure 4a, the response intensities of the 10 sensors varied markedly, reflecting significant changes in the volatile profiles of braised pork during processing. The total response value after processing was significantly higher than that of the raw meat. This indicated that thermal treatment promoted the release of volatile compounds from the meat, leading to greater diversity and higher concentrations.

Throughout all processing stages of braised pork, sensors W5S, W1S, W2S, and W1W exhibited consistently high response values, suggesting relatively high levels of nitrogen oxides, alkanes, alcohols and aromatic compounds, terpenes, and sulfur-containing organic compounds in the samples. Among these, the response values of sensors W1S and W5S were particularly pronounced and increased continuously with longer processing times. In contrast, sensors W5C, W3C, and W1C exhibited markedly lower response values throughout the process. This was primarily due to the generally high odor thresholds of their target substances (aliphatic hydrocarbons), which results in a weaker sensor response [56]. Specifically, the response of the W5S sensor, which is sensitive to nitrogen oxides, was significantly higher during stewing processing stages (B0–B3) compared to raw meat. This suggested the substantial generation of nitrogen oxides during the stewing process. These compounds are important contributors to a meaty aroma and are primarily generated through protein oxidation reactions. However, this pronounced change was not directly detected in the GC-MS analysis. The discrepancy likely arises from the fundamental difference between the two techniques: the E-nose provides a holistic aroma profile, whereas GC–MS focuses on quantifying individual volatile compounds [57].

Principal component analysis (PCA) was applied to the E-nose data for unsupervised dimensionality reduction, providing a more intuitive and comprehensive visualization of the evolution of aroma characteristics in braised pork across different processing stages as shown in the Figure 4b. The first two principal components (PC1 and PC2) cumulatively accounted for 86.2% of the total variance (67.2% and 19.0%, respectively). In the PCA score plot, samples from the five distinct processing stages were clearly distinguishable, despite minor overlap among a few individual data points. This distribution pattern indicated a progressive transformation of odor characteristics during thermal processing, with each stage developing differentiated aromatic qualities and forming separable characteristic clusters.

### 3.9. Analysis of Free Amino Acids and Nucleotide in Flavor Components

**Figure 5:**
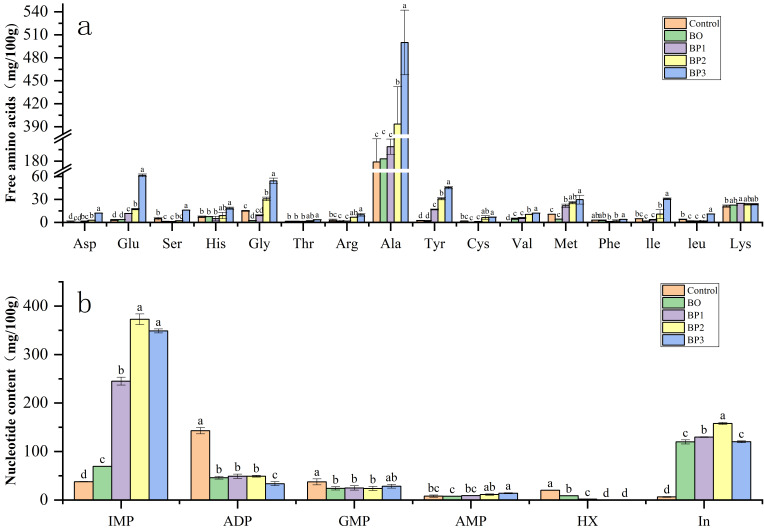
Amino acids (**a**) and nucleotides (**b**). Data are expressed as the mean ± standard deviation (n = 3); different letters within the same row indicate significant differences among treatments (*p* < 0.05), as determined via one-way analysis of variance (ANOVA) followed by Tukey’s multiple comparison test.

Flavor development is closely associated with non-volatile compounds, particularly nucleotides and free amino acids (FAAs) [58]. In braised pork, FAAs are primarily generated through the proteolytic degradation of proteins: endogenous proteases first cleave proteins into short peptides, which are subsequently hydrolyzed into free amino acids [59]. Free amino acids (FAAs) possessed distinct sensory attributes. Aspartic acid and glutamic acid were responsible for the umami taste, while sweet notes were attributed to glycine, alanine, serine, threonine, and proline. Several other FAAs, including methionine, leucine, isoleucine, histidine, arginine, and phenylalanine, imparted bitter notes. Collectively, these FAAs were pivotal in defining the characteristic flavor profile of braised pork [60]. As shown in Figure 5b, the contents of free amino acids changed significantly throughout the processing stages of braised pork. The total FAA content in raw meat was 262.01 mg/100 g, which increased to 838.67 mg/100 g in the final product. Notably, the umami amino acids Asp, Glu along with sweet amino acid Ala, exhibited a particularly marked increase (*p* < 0.05). This rise can be attributed to prolonged heating during stewing, which promotes continuous protein breakdown and the consequent accumulation of FAAs. The enhanced levels of these key taste-active amino acids were consistent with the overall flavor improvement of braised pork after thermal processing. In meat flavor systems, Glu not only strongly contributes to umami perception but also synergizes with chloride ions to produce a monosodium-glutamate-like savory sensation [61]. Furthermore, Asp and Glu exhibit significant synergistic enhancement with 5′-ribonucleotides, particularly 5′-inosine monophosphate (IMP) and 5′-adenosine monophosphate (AMP) [62].

Free 5′-nucleotides are crucial for flavor perception in meat and its products, due to their ability to impart a pronounced umami character [63]. As shown in the Figure 5a, free 5′-nucleotide components, including AMP, IMP, GMP, ADP, hypoxanthine (Hx), and inosine (In), were detected in braised pork. Among these compounds, IMP exhibited a clear increasing trend with an extended stewing time, whereas other nucleotides and degradation products showed different variation patterns during processing. The contents of umami nucleotides, predominantly IMP, showed an increasing trend during heating, which may have been a key factor contributing to the superior final flavor of braised pork. Compared with raw meat, the concentration of umami nucleotides increased significantly in the processed meat (*p* < 0.05). This increase was primarily attributed to the thermal activation of phosphatases, which drove a cascade of degradation reactions: adenosine triphosphate (ATP) in the meat is converted to adenosine diphosphate (ADP) by ATPase, ADP is then transformed to AMP by adenylate kinase, and AMP is subsequently deaminated to yield IMP. IMP can be further partially converted to inosine by nucleotidase and subsequently hydrolyzed to hypoxanthine [64]. Additionally, the thermal degradation of IMP to inosine during heating also contributes to the enhancement of meat flavor [43]. Among the flavor-active nucleotides, 5′-AMP, 5′-IMP, and 5′-GMP are particularly important. Their synergistic interactions with glutamate derivatives can significantly enhance umami perception and collectively shape the characteristic meaty flavor quality [65].

## 4. Conclusions

This study systematically elucidated the integrated mechanisms governing flavor and quality development in braised pork during stewing via multiple analytical techniques. Results revealed that thermal processing regulated the texture and flavor through interconnected reactions. Tenderness development followed a pattern of an initial decrease due to protein denaturation, followed by a fundamental improvement driven by collagen gelatinization and tissue degradation. Flavor complexity increased progressively, with total volatile compounds rising from 11 to 33 and key lipid oxidation markers (aldehydes, ketones) accumulating continuously. Protein oxidation (increased carbonyls, decreased sulfhydryl groups) and water redistribution toward free states were identified as interconnected processes co-regulating both texture and flavor formation. Furthermore, stewing enhanced umami perception through the proteolytic release of free amino acids (Asp, Glu) and thermal-driven nucleotide catabolism, particularly IMP accumulation. The electronic nose and PCA confirmed distinct stage-specific aroma profiles. Overall, these findings demonstrate that the improvement in eating quality of braised pork during stewing is closely associated with measurable changes in texture, water distribution, oxidation-related indices, and flavor-active compounds. This study provides experimentally supported insights into quality formation during braising and offers scientific support for the standardization and industrial optimization of traditional braised pork processing.

## Figures and Tables

**Table 1:** Texture profile of lean meat during the cooking of braised pork.

	Different Processing Stages				
	Control	BO	BP1	BP2	BP3
Hardness	82.25 ± 14.66 ^d^	455.03 ± 47.8 ^a^	393.6 ± 26.08 ^b^	183.61 ± 42.97 ^c^	130.58 ± 17.1 ^cd^
Chewiness	34.44 ± 14.68 ^d^	233.78 ± 43.65 ^a^	173.79 ± 15.52 ^b^	79.06 ± 5.47 ^c^	57.35 ± 22.11 ^cd^
Elasticity	0.49 ± 0.04 ^c^	0.79 ± 0.05 ^a^	0.74 ± 0.07 ^a^	0.67 ± 0.12 ^bc^	0.54 ± 0.19 ^bc^
Cohesiveness	0.5 ± 0.1 b^c^	0.71 ± 0.04 ^a^	0.58 ± 0.06 ^ab^	0.53 ± 0.06 ^b^	0.38 ± 0.2 ^c^
Springiness	0.3 ± 0.12 ^b^	0.63 ± 0.03 ^a^	0.23 ± 0.03 ^bc^	0.21 ± 0.04 ^bc^	0.14 ± 0.07 ^c^
Adhesiveness	7.63 ± 2.22 ^d^	12.11 ± 0.52 ^c^	24.98 ± 3.23 ^a^	27.47 ± 1.91 ^a^	20.04 ±1.59 ^b^
Shear force	1225.6 ± 215.03 ^ab^	1432.65 ± 31.01 ^a^	1065.38 ± 124.88 ^bc^	856.24 ± 35.51 ^cd^	610.94 ± 77.58 ^d^

Different letters on the same row indicate significant differences (*p* < 0.05), as determined via one-way analysis of variance (ANOVA) followed by Tukey’s multiple comparison test.

## Data Availability

The original contributions presented in the study are included in the article; further inquiries can be directed to the corresponding author.

## Appendix A

**Table A1:** Volatile flavor compounds.

Volatile Compounds (µg/kg)	Processing Stage				
	Control	BO	BP1	BP2	BP3
**Aldehydes**					
Hexanal	0.41 ± 0.05 ^d^	179.23 ± 2.73 ^c^	226.62 ± 4.06 ^b^	312.06 ± 13.31 ^a^	353.34 ± 11.30 ^a^
Nonanal	55.13 ± 1.50 ^d^	74.22 ± 4.80 ^c^	112.79 ± 7.60 ^b^	90.17 ± 5.40 ^c^	130.53 ± 5.00 ^a^
Heptanal	0	36.84 ± 2.00 ^b^	114.20 ± 8.00 ^a^	29.70 ± 1.50 ^c^	2.70 ± 0.20 ^d^
Octanal	0	15.62 ± 1.00 ^a^	0	0	0
2,4-Decadienal	0	3.51 ± 0.40 ^d^	75.37 ± 2.00 ^b^	43.37 ± 3.00 ^c^	92.83 ± 5.00 ^a^
(E)-2-Octenal	0	25.29 ± 0.20 ^a^	11.03 ± 0.50 ^c^	8.99 ± 0.01 ^d^	13.71 ± 0.01 ^b^
2,6-Octanedione	0	0	147.00 ± 5.00 ^b^	141.80 ± 8.00 ^b^	217.27 ± 10.00 ^a^
Benzaldehyde	0	0	0.07 ± 0.01 ^c^	2.44 ± 0.10 ^a^	1.37 ± 0.10 ^b^
Nerolidol	0	0	72.23 ± 5.00 ^b^	61.00 ± 5.00 ^c^	91.67 ± 5.00 ^a^
**Alcohols**					
(E)-2-Decen-1-ol	18.34 ± 0.50 ^a^	0	0	0	0
1-Octen-3-ol	0	37.38 ± 0.50 ^a^	11.56 ± 0.10 ^d^	11.84 ± 0.10 ^c^	12.52 ± 0.10 ^b^
1-Pentanol	0	9.72 ± 0.10 ^a^	1.04 ± 0.01 ^d^	4.31 ± 0.10 ^b^	3.66 ± 0.10 ^c^
5-Methyl-1-heptanol	0	1.90 ± 0.10 ^a^	0	0	0
4-Heptanol	0	0	0	3.04 ± 0.10 ^b^	4.79 ± 0.10 ^a^
4-Methyl-1-pentanol	0	0	1.43 ± 0.10 ^a^	0.11 ± 0.01 ^b^	0
2-Nonen-1-ol	0	0	0.08 ± 0.01 ^c^	10.79 ± 0.10 ^b^	24.83 ± 0.10 ^a^
Eucalyptol	0	0	4.54 ± 0.10 ^a^	2.06 ± 0.10 ^b^	0
Alpha-terpineol	0	0	0.41 ± 0.01 ^b^	0.10 ± 0.01 ^c^	5.68 ± 0.10 ^a^
2,5-Dimethyl-1,5-hexadien-3-ol	0	0	0	0	0.09 ± 0.01 ^a^
2-Methyl-2-undecanethiol	0	0	10.06 ± 0.10 ^a^	0	0
**Acids**					
Octanoic acid	0	0	19.73 ± 0.10 ^b^	14.86 ± 0.10 ^c^	34.70 ± 0.10 ^a^
Pentanoic acid	57.31 ± 0.10 ^a^	0	24.14 ± 0.10 ^b^	0	7.74 ± 0.10 ^c^
2-Pentyl-4-ethylbenzoic acid	0	29.00 ± 0.10 ^c^	0	29.54 ± 0.10 ^b^	25.35 ± 0.10 ^c^
2-Methylheptanoic acid	0.12 ± 0.01 ^a^	0	0	0	0
2-Methylhexanoic acid	1.05 ± 0.10 ^a^	0	0	0	0
2-Butyl ester phenylacetic acid	0	0	0.36 ± 0.01 ^a^	0	0
**Ketones**					
2,3-Octanedione	0	37.12 ± 0.10 ^a^	24.14 ± 0.10 ^b^	0.58 ± 0.01 ^d^	7.74 ± 0.10 ^c^
3-Ethyl-2-pentanone	0	1.83 ± 0.10 ^c^	0	39.48 ± 0.10 ^a^	33.45 ± 0.10 ^b^
4-Hydroxycyclohexanone	0	0.93 ± 0.01 ^d^	147.00 ± 5.00 ^b^	141.80 ± 8.00 ^b^	217.27 ± 10.00 ^a^
**Esters**					
Trans-2-Hexenyl isobutyrate	0	6.68 ± 0.10 ^c^	20.19 ± 0.10 ^a^	13.05 ± 0.10 ^b^	3.71 ± 0.10 ^d^
Methyl heptanoate	0.97 ± 0.01 ^a^	0	0	0	0
Methyl butyrate	0	0	0.29 ± 0.01 ^a^	0.11 ± 0.01 ^b^	0
Cyclohexyl ester	0	0	0.30 ± 0.01 ^a^	0.11 ± 0.01 ^b^	0
Methyl hexanoate	0	0	0.27 ± 0.01 ^a^	0	0
S-Methyl hexanethioate	0	0	19.23 ± 0.10 ^a^	1.17 ± 0.10 ^b^	0
Propyl butyrate	0	0	0	0.27 ± 0.01 ^b^	1.06 ± 0.10 ^a^
**Olefins**					
1-Tetradecene	0	5.72 ± 0.10 ^d^	67.51 ± 0.10 ^b^	56.70 ± 0.10 ^c^	126.38 ± 0.10 ^a^
6-Methyl-1-heptene	0.77 ± 0.01 ^a^	0	0	0	0
Cyclohexene 3-(tert-butyl) peroxide	0	0.75 ± 0.01 ^d^	2.55 ± 0.10 ^c^	1.37 ± 0.10 ^d^	9.45 ± 0.10 ^a^
4-Methyl-1-decene	0	0	0.40 ± 0.01 ^a^	0	0
4,5-Dimethyl-1-hexene	0	0	0.15 ± 0.01 ^a^	0	0
1-Octene	0	0	0.44 ± 0.01 ^c^	1.11 ± 0.10 ^b^	6.81 ± 0.10 ^a^
Beta-lauroene	0	0	0	0	3.52 ± 0.10 ^a^
Camphene	0	0	0	0	0.54 ± 0.01 ^a^
Alpha-pinene	0	0	0	0	0
**Alkanes**					
3-Methylhexane	0	2.14 ± 0.10 ^b^	0	0	5.22 ± 0.10 ^a^
4-Methyldecane	0	0	0.13 ± 0.01 ^a^	0	0
2,2,4,6,6-Pentamethylheptane	0	0	0.64 ± 0.01 ^b^	0	1.03 ± 0.10 ^a^
5-Methyltridecane	0	0	0	0	0
Tridecane	1.53 ± 0.10 ^b^	14.60 ± 0.10 ^a^	0.94 ± 0.01 ^c^	0	0.17 ± 0.01 ^d^
Undecane	0.85 ± 0.01 ^d^	6.06 ± 0.10 ^b^	0.07 ± 0.01 e	0.56 ± 0.01 ^d^	2.50 ± 0.10 ^a^
2,3-Dimethylundecane	0	0	0.25 ± 0.01 ^a^	0	0
Pentylcyclopropane	0.99 ± 0.01 ^a^	0	0	0	0
Decane	0	5.01 ± 0.10 ^a^	0	0.17 ± 0.01 ^b^	0
**Heterocycles**					
5-Methyl-1-hexyne	0	0	2.32 ± 0.10 ^a^	0.90 ± 0.01 ^b^	0
3-Ethyl-2,5-dimethylpyrazine	0	0	3.5 ± 1.15 ^c^	12.3 ± 2.01 ^b^	14.6 ± 1.47 ^a^
Endo-borneol	0	0	0.30 ± 0.01 ^a^	0	0.12 ± 0.01 ^b^
Naphthalene	0.20 ± 0.01 ^a^	0	0	0	0
2-Pentylfuran	0	20.01 ± 0.10 ^a^	0	0	0
2-Butylamine	0	4.08 ± 0.10 ^a^	0	0	0

For each volatile compound, different letters within the same row indicate significant differences among treatments (*p* < 0.05), as determined via one-way analysis of variance (ANOVA) followed by Tukey’s multiple comparison test.
